# CRISPR-cas9 Screening Identified Lethal Genes Enriched in Cell Cycle Pathway and of Prognosis Significance in Breast Cancer

**DOI:** 10.3389/fcell.2021.646774

**Published:** 2021-03-19

**Authors:** Xi Sun, Zheng Wang, Xiaosong Chen, Kunwei Shen

**Affiliations:** Department of General Surgery, Comprehensive Breast Health Center, Ruijin Hospital, Shanghai Jiao Tong University School of Medicine, Shanghai, China

**Keywords:** CRISPR-cas9 screening, breast cancer, cell cycle, signature, cell viability

## Abstract

**Background:**

Lethal genes have not been systematically analyzed in breast cancer which may have significant prognostic value. The current study aims to investigate vital genes related to cell viability by analyzing the CRISPR-cas9 screening data, which may provide novel therapeutic target for patients.

**Methods:**

Genes differentially expressed between tumor and normal tissue from the Cancer Genome Atlas (TCGA) and genes related to cell viability by CRISPR-cas9 screening from Depmap (Cancer Dependency Map) were overlapped. Kyoto Encyclopedia of Genes and Genomes (KEGG) and Gene Ontology (GO) analysis was conducted to identify which pathways of overlapped genes were enriched. GSE21653 set was randomized into training and internal validation dataset at a ratio of 3:1, and external validation was performed in GSE20685 set. The least absolute shrinkage and selection operator (LASSO) regression was used to construct a signature to predict recurrence-free survival (RFS) of breast cancer patients. Univariate and multivariate Cox regression were used to evaluate the prognostic value of this signature. Differentially expressed genes (DEGs) between high-risk and low-risk patients were then analyzed to identify the main pathways regulated by this signature. Weighted correlation network analysis (WGCNA) was conducted to recognize modules correlated with high risk. Enrichment analysis was then used to identify pathways regulated by genes shared in the overlapped genes, DEGs, and WGCNA.

**Results:**

A total of 86 oncogenes were upregulated in TCGA database and overlapped with lethal genes in Depmap database, which were enriched in cell cycle pathway. A total of 51 genes were included in the gene signature based on LASSO regression, and the median risk score of 2.36 was used as cut-off to separate low-risk patients from high-risk patients. High-risk patients showed worse RFS compared with low-risk patients in internal training, internal validation, and external validation dataset. Time-dependent receiver operating characteristic curves of 3 and 5 years indicated that risk score was superior to tumor stage, age, and PAM50 in both entire and external validation datasets. Cell cycle was the main different pathway between the high-risk and low-risk groups. Meanwhile, cell cycle was also the main pathway enriched in the 25 genes which were shared among 86 genes, DEGs, and WGCNA.

**Conclusion:**

Cell cycle pathway, identified by CRISPR-cas9 screening, was a key pathway regulating cell viability, which has significant prognostic values and can serve as a new target for breast cancer patient treatment.

## Introduction

Breast cancer, with the highest incidence rate among female cancer, is the second leading cause of cancer-related death and poses a great threat to women’s health (Siegel et al., 2020). It accounts for 30% of all new cases and is responsible for 15% of cancer deaths in women. Despite that the prognosis of patients has been improving over the past few years, the complex biological behaviors of breast cancer still hamper the progress in clinical treatment. Thus, understanding the specific vulnerability of breast carcinoma is of great importance.

Currently, CRISPR-cas9 screening is emerging as a powerful tool for precise medicine (Doudna and Charpentier, 2014; Kurata et al., 2018). Combining cas9 with pooled guide RNA libraries facilitates screening of genes that contribute to specific biologic phenotypes and diseases in a high-throughput way (Joung et al., 2017). This “phenotype-to-genotype” approach includes modifying expression of genes, selecting cells with a phenotype of interest, and sequencing the perturbation of interest, which allows for discovering genes related to cell viability (Schuster et al., 2019). Meanwhile, large-scale loss-of-function screening for cancer dependences have been performed in a variety of well-characterized cancer cell lines to assess the effect of single-gene knockout on cell viability (Meyers et al., 2017; Tsherniak et al., 2017). These data were deposited in the Cancer Dependence Map (DepMap) website.

Aberrant cell cycle is a hallmark of cancer (Hanahan and Weinberg, 2011). The evolution of cell cycle is conservative. Checkpoints have evolved to ensure that cell cycle progress is under sequential activation (Kastan and Bartek, 2004; Strzyz, 2016). Under the stimulation of mitogenic signal, cyclin-dependent kinases (CDKs) associate with cyclins and phosphorylate intracellular proteins that orchestrate cell cycle progress in a well-organized way (Malumbres and Barbacid, 2009; Malumbres, 2014). In cancer cells, aberrant signals are developed and promote the activation of CDK–cyclin complex. Deregulation of the cell cycle engine eventually leads to uncontrolled cell proliferation and genomic instability in cancer. Thus, the therapeutic potential of targeting the cell cycle has been increasingly concerned (Lim and Kaldis, 2013). In breast cancer, the application of CDK4/6 inhibitor transformed treatment landscape in estrogen (ER)-positive human epidermal growth factor receptor-2 (HER2) negative breast cancer. The improvement in prognosis indicated that targeting cell cycle is an essential way to cancer treatment (Ingham and Schwartz, 2017; Slamon et al., 2018; Tripathy et al., 2018).

Biological process involving cell viability is complex. However, cell vulnerability of breast cancer has not been systematically researched. Meanwhile, pathways and the prognostic significance of these genes have never been detailed. In the current study, we aimed to identify genes differentially expressed in tumor tissues and contributed to cell viability. Using these genes, a prediction model with prognostic significance was constructed and validated. The pathways and biological processes regulated by these genes were also evaluated.

## Materials and Methods

### Identification of Viability Vulnerable

Dependence scores of breast cancer cell lines were downloaded from the Depmap dataset^1^, and this is the result from a series of loss-of-function genomic screening in different cell lines. Dependence score was calculated by CERES algorithm to identify genes essential to proliferation and survival (Meyers et al., 2017). A negative score of a gene indicates that knocking out of the gene inhibits the survival of a cell line, whereas a positive score indicates that knocking out of the gene promotes the survival and proliferation. Cut-offs of 0.5 and −0.5 were to define growth-suppressing genes and growth-promoting genes.

Read counts of breast cancer were downloaded from TCGA datasets^2^. Differentially expressed genes between tumor and normal patients were calculated based on the negative binomial distribution using DESeq2 package. Adjusted *p* value <0.05 and absolute fold change greater than 2 were used as cut-off to select differentially expressed genes. Growth-suppressing genes from Depmap were overlapped with downregulated genes from the TCGA dataset, and growth-promoting genes from Depmap were overlapped with upregulated genes from the TCGA dataset to select genes for further analysis.

### Data Processing

Raw data of GSE20685 and GSE21653 were downloaded from the GEO database^3^. These two datasets were both from [HG-U133_Plus_2] Affymetrix Human Genome U133 Plus 2.0 platform. The raw data of GSE20685 and GSE21653 were normalized by gcrma algorithm simultaneously. The probe ID was converted into gene symbol using the annotation platform. When one probe was matched to the same gene, average gene expression of this gene was calculated.

### LASSO Regression Analysis

Patients from GSE21653 set were randomized into internal training dataset and internal validation dataset at a ratio of 3:1. The least absolute shrinkage and selection operator (LASSO) model was used to remove genes of high correlation and a risk model was constructed (Tibshirani, 1997; Zhou et al., 2019). A risk score formula was established by integrating gene expression value weighted by their LASSO Cox coefficients. R package “glmnet” in R 3.5.2 was used to perform LASSO analysis (Ternès et al., 2016). Univariate and multivariate Cox regression analysis was used to assess the prognostic value of risk score in entire dataset and external validation dataset. Time-dependent receiver operating characteristic (tROC) curves were used to compare the prediction accuracy of risk score with traditional clinicopathological parameters. “survivalROC” package was used to plot tROC curve and calculate Area under curve (AUC).

### Differentially Expressed Genes

In dataset GSE21653, limma package was used to calculate differentially expressed genes between high-risk group and low-risk group patients. *P* value <0.05 and absolute fold change greater than 1.5 were defined as DEGs.

### Enrichment Analysis

Gene Ontology (GO) was used to annotate biological processes, molecular functions, and cellular components of genes. Kyoto Encyclopedia of Genes and Genomes (KEGG) was used to annotate the gene pathways (Gene Ontology Consortium, 2015; Kanehisa et al., 2017). GO and KEGG analysis was performed using clusterProfiler package (Yu et al., 2012). *P* value <0.05 was considered as significant pathways enriched.

### PPI Network Construction and Hub Gene Identification

STRING^4^ website was used to discover known and predicted protein–protein interactions, as well as to construct a PPI network. The Cytoscape software was then employed to visualize the interactive relationship of the overlapped genes.

### Weighted Correlation Network Analysis

Weighted Correlation Network Analysis (WGCNA) was performed to find modules of highly correlated genes using WGCNA package (Langfelder and Horvath, 2008). A One-step network construction was used to construct network and modules were identified. Eigengenes were correlated with external traits to identify modules that are significantly associated with the measured clinical traits. A scatterplot of Gene Significance (GS) versus Module Membership (MM) in different modules was plotted to show the correlation of GS and MM.

### Statistical Analysis

Survival analysis was evaluated using Kaplan–Meier analysis with the log-rank test. *P* value <0.05 was defined as statistically significant. The time-dependent AUC value was calculated by the survivalROC package.

## Results

### Identification of Functional Genomic Genes in Breast Cancer

**FIGURE 1:**
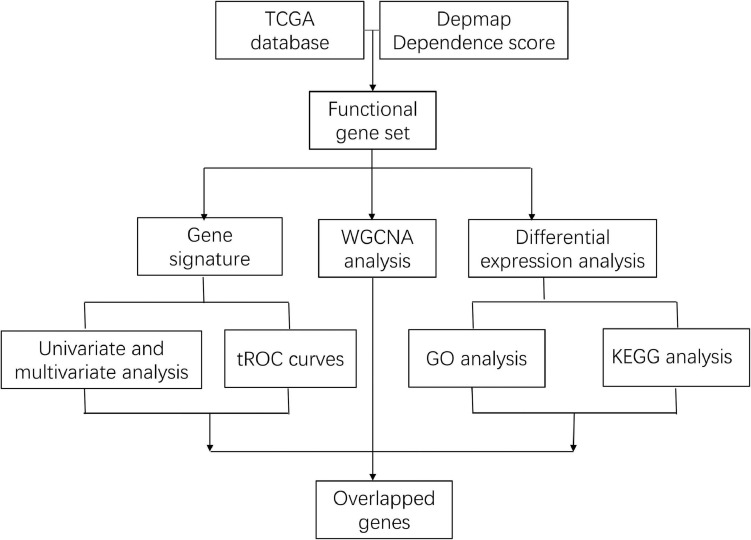
Flowchart of the entire analysis.

**FIGURE 2:**
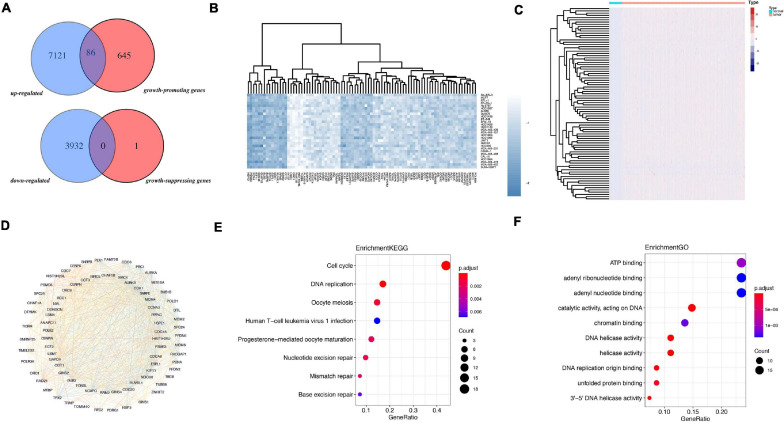
Identification of oncogenes from TCGA and Depmap dataset. **(A)** Overlapped genes between TCGA and Depmap dataset. **(B)** The dependence score of 86 oncogenes in breast cancer cell lines. **(C)** The differential expression of the 86 genes in TCGA dataset between tumor and normal. **(D)** The PPI network of the 86 genes. **(E)** KEGG analysis of 86 genes. **(F)** GO analysis of 86 genes.

The flowchart of analysis is shown in Figure 1. A total of 28 cell lines of breast cancer have dependence scores on the Depmap website. Genes with dependence score less than −0.5 in all breast cancer cell lines were overlapped with upregulated genes in TCGA and 86 genes were discovered. Meanwhile, genes with dependence score greater than 0.5 in all breast cancer cell lines were overlapped with downregulated genes in TCGA and none of the genes were overlapped (Figure 2A). These 86 genes were defined as oncogenes. The dependence scores of oncogenes are shown in Figure 2B and expression of these genes in TCGA are shown in Figure 2C. PPI network revealed there contains 1117 interactions among these proteins (Figure 2D). Overall, the mutation rate of these genes was low, and INTS7 had the highest mutation rate of 0.8% (Supplementary Figure 1). KEGG analysis demonstrated that these oncogenes were enriched in pathways including cell cycle, DNA replication, oocyte meiosis, and nucleotide excision repair gene (Figure 2E). In GO analysis, the top three pathways enriched were ATP binding, adenyl ribonucleotide binding, and adenyl nucleotide binding (Figure 2F).

### Gene Signature Construction and Validation

**FIGURE 3:**
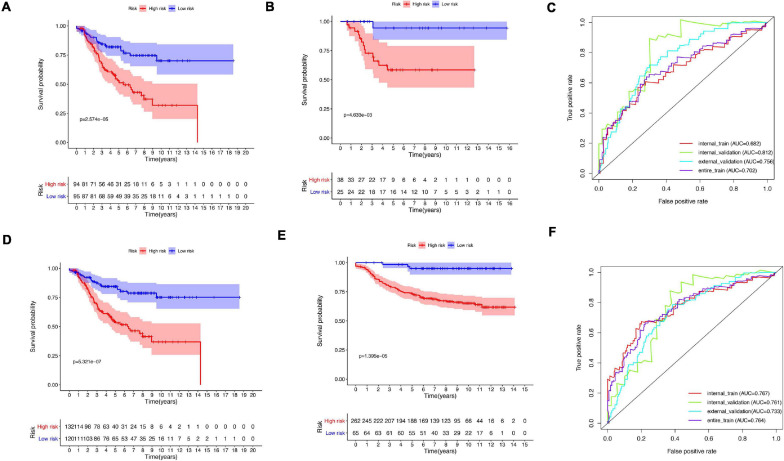
Kaplan–Meier plot and AUC curve of discovery and validation cohort based on gene signature. **(A)** Kaplan–Meier plot of the internal train cohort. **(B)** Kaplan–Meier plot of the internal validation cohort. **(C)** AUC area of tROC curve of 3-year survival. **(D)** Kaplan–Meier plot of the entire cohort. **(E)** Kaplan–Meier plot of the external validation cohort. **(F)** AUC area of tROC curve of 5-year survival.

**FIGURE 4:**
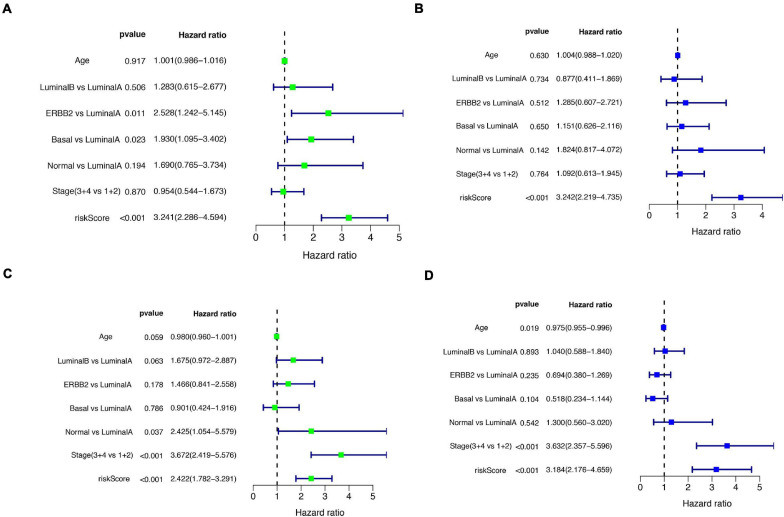
Univariate and multivariate Cox regression analyses of the entire train and validation cohort. **(A)** Univariate Cox regression analysis in the entire cohort. **(B)** Parameters significant in univariate Cox regression were included in multivariate Cox regression analysis in the entire cohort. **(C)** Univariate Cox regression analysis in the external validation cohort. **(D)** Parameters significant in univariate Cox regression were included in multivariate Cox regression analysis in external validation cohort.

**FIGURE 5:**
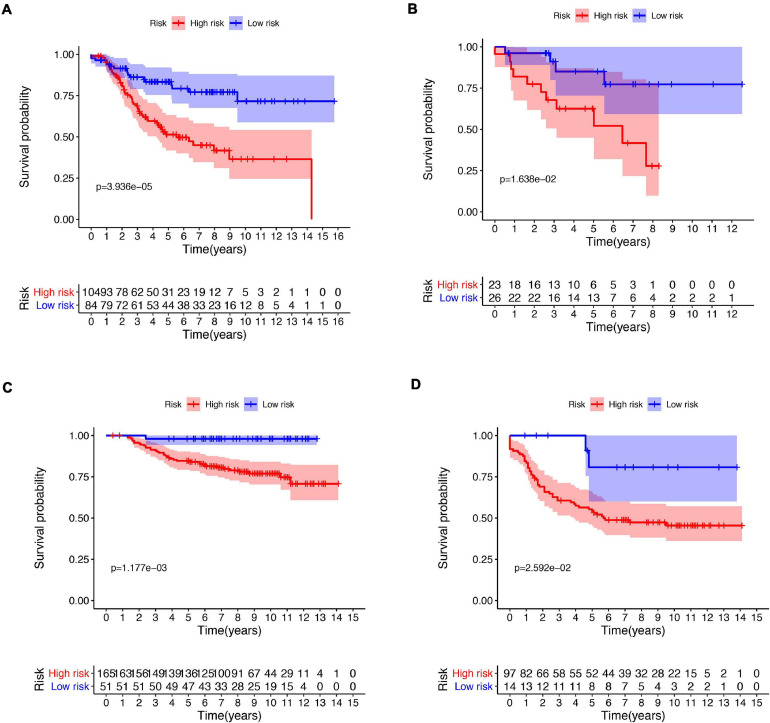
Kaplan–Meier plot of risk score in different subtypes. Kaplan–Meier plot of risk score in stage I–II **(A)** and stage III–IV **(B)** in the entire dataset. Kaplan–Meier plot of risk score in stage I–II **(C)** and stage III–IV **(D)** in external validation dataset.

**FIGURE 6:**
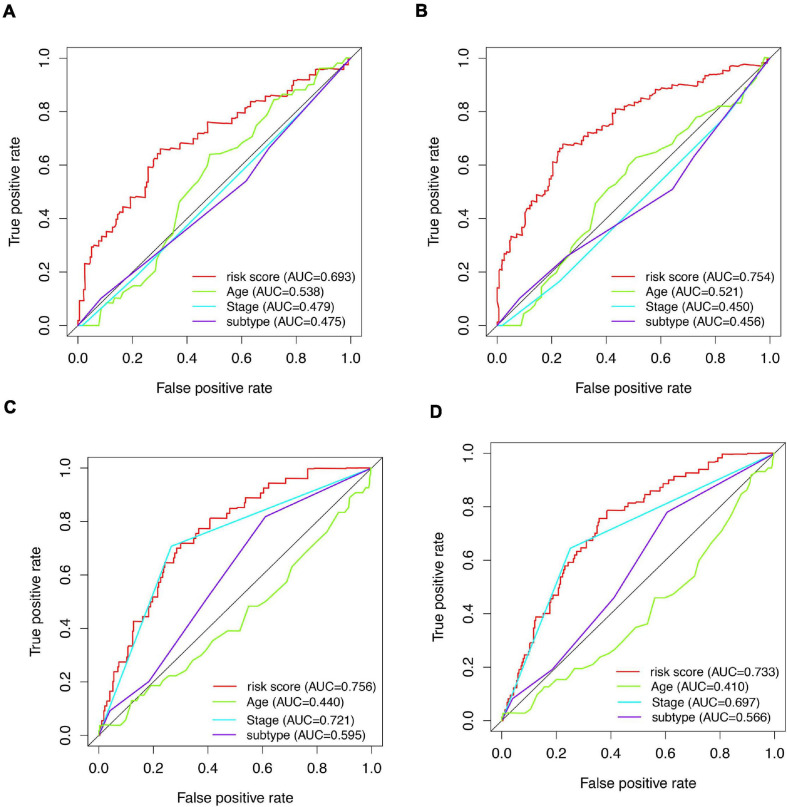
AUCs of tROC curve for clinicopathologic parameters and risk score. **(A)** tROC curves of 3-year survival for clinicopathologic parameters and risk score in the entire train dataset. **(B)** tROC curves of 5-year survival for clinicopathologic parameters and risk score in the entire train dataset. **(C)** tROC curves of 3-year survival for clinicopathologic parameters and risk score in the validation dataset. **(D)** tROC curves of 5-year survival for clinicopathologic parameters and risk score in the validation dataset.

A total of 51 genes were screened out by Lasso regression model, and a risk formula was constructed. The coefficients are listed in Supplementary Table 1. The median risk score 2.36 was used as cut-off value to divide patients into high-risk and low-risk group in internal training dataset and the same cut-off was also used in validation dataset. Patients from the high-risk group had significantly shorter median RFS of 5.52 years compared with patients from the low-risk group in internal training dataset (Figure 3A, *p* < 0.001, HR: 2.88, 95% CI: 1.72–4.82). This was validated in internal and external validation dataset. As expected, patients with high risk had poorer RFS compared with patients in low-risk group in internal validation dataset (Figure 3B, *p* < 0.001, HR: 10.54, 95% CI:1.38–80.79), entire train dataset (Figure 3D, *p* < 0.001, HR: 3.28, 95% CI: 2.00–5.36), and external validation dataset (Figure 3E, *p* < 0.001, HR: 8.39, 95% CI: 2.65–26.48). Cut-off calculated by ROC curve was used and 2.68 was used as a cut-off to separate patients into different risks. Similarly, both in the entire dataset and validation dataset, there was statistical significance between high-risk and low-risk groups (Supplementary Figure 2). For 3-year tROC curves, AUC area was 0.682, 0.812, 0.702, and 0.756 for internal train, internal validation, entire train, and external validation dataset (Figure 3C). For 5-year tROC curves, AUC area was 0.767, 0.761,0.764, and 0.733 for internal train, internal validation, entire train, and external validation dataset, respectively (Figure 3F). The distribution of risk scores of different risk groups are shown in risk plots (Supplementary Figure 3). After adjustment of clinicopathological variables, Cox regression demonstrated that the risk score was an independent prognostic signature in the entire train set (Figures 4A,B, *p* < 0.001, HR: 3.24, 95% CI: 2.22–4.74) and external validation set (Figures 4C,D, *p* < 0.001, HR: 3.18, 95% CI: 2.18–4.66). Stratified analysis suggested that the signature was clinically significant in stage I + II, stage III + IV cases (Figures 5A–D). In both luminal and HER2 amplification groups, RFS of the high-risk group was also worse than that of the low-risk group in validation dataset (Supplementary Figure 4, luminal group: *p* < 0.0002, HER2 amplification group: *p* = 0.02). A total of 45 patients were included in the basal group and only one patient was included in the low-risk group, so basal cohort with more patients were needed to validate the performance of this signature (*p* = 0.6). Stratified analysis was done in dataset GSE20685 between patients with chemotherapy and without chemotherapy. In patients receiving chemotherapy, RFS of high-risk group was worse than that of low-risk group (Supplementary Figure 5, *p* < 0.0003). In patients without therapy, the RFS of high-risk group was worse than that of low-risk group, but there was no statistical significance (*p* = 0.1). Meanwhile, our signature outperformed age, stage, and PAM50 subtype in both datasets (Figure 6). For 3-year tROC curves, AUC area was 0.693, 0.538, 0.479, and 0.475 for risk score, age, stage, and PAM50 subtype in entire dataset (Figure 6A). For 5-year tROC curves, AUC area was 0.754, 0.521, 0.450, and 0.456 for risk score, age, stage, and PAM50 subtype in entire dataset (Figure 6B). For 3-year tROC curves, AUC area was 0.756, 0.440, 0.721, and 0.596 for risk score, age, stage, and PAM50 subtype in external validation dataset (Figure 6C). For 5-year tROC curves, AUC area was 0.733, 0.410, 0.697, and 0.566 for risk score, age, stage, and PAM50 subtype in external validation dataset (Figure 6D).

### DEGs Between High-Risk and Low-Risk Groups

**FIGURE 7:**
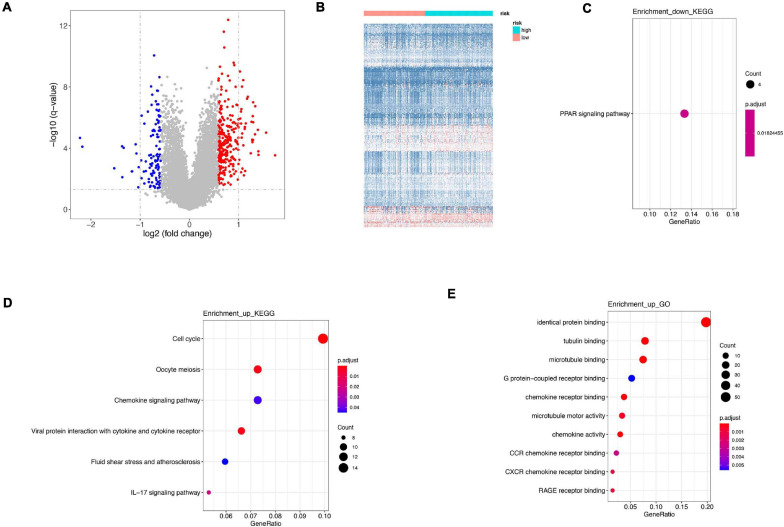
Difference between high risk and low risk. **(A)** Differentially expressed genes in high-risk and low-risk group. **(B)** Heatmap of differentially expressed genes in high-risk and low-risk group. **(C)** KEGG analysis of downregulated genes. **(D)** KEGG analysis of upregulated genes. **(E)** GO analysis of upregulated genes.

Differentially expressed genes were calculated between high-risk and low-risk groups in GSE21653 dataset. A total of 398 were identified, among which 285 genes were upregulated and 113 genes were downregulated (Figures 7A,B). Downregulated genes were only enriched in PPAR signaling pathway (Figure 7C), whereas upregulated genes were involved in cell cycle, oocyte meiosis, chemokine signaling pathway in KEGG analysis, and in identical protein binding, tubulin binding, and microtubule binding in GO analysis (Figures 7D,E). Interestingly, both in our signature and DEGs, cell cycle and oocyte meiosis were both enriched, indicating cell cycle was an important way regulating cell viability.

### WGCNA

**FIGURE 8:**
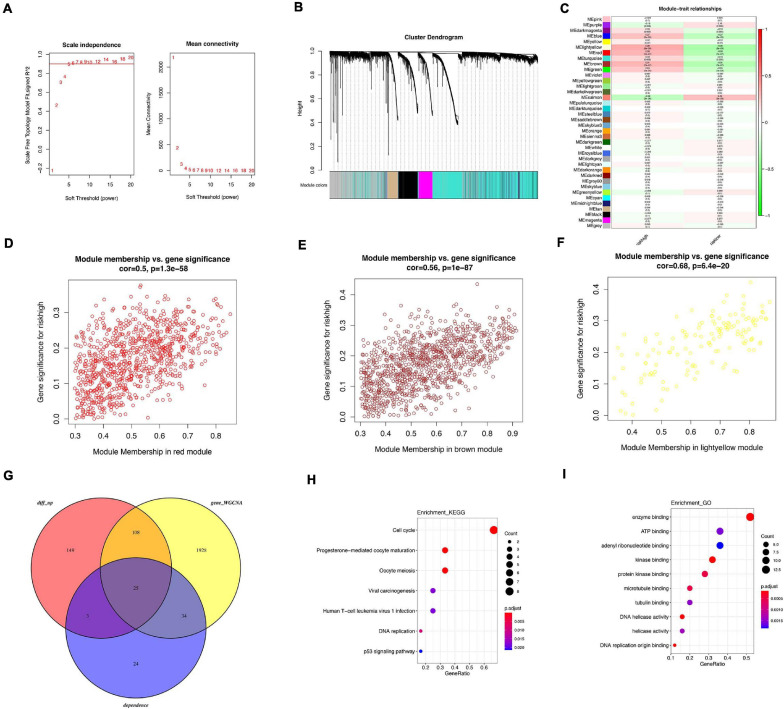
WGCNA analysis related with high risk. **(A)** Analysis of scale-free fit index for various soft threshold powers. **(B)** Co-expression network modules for mRNA. **(C)** Heatmap of trait correlation with tamoxifen resistance. **(D)** Scatter plots of module membership in red module and gene significance. **(E)** Scatter plots of module membership in brown module and gene significance. **(F)** Scatter plots of module membership in light yellow module and gene significance. **(G)** Venn diagram of overlapped genes among 86 oncogenes, DEGs between high-risk and low-risk patients and WGCNA modules. **(H)** KEGG analysis of overlapped genes. **(I)** GO analysis of overlapped genes.

WGCNA was performed in GSE21653 dataset to identify hub genes. Soft threshold power of 5 was selected to ensure a scale-free network (Figure 8A). A total of 37 clusters were clustered using cut height 0.25 (Figure 8B). Several modules were correlated with high-risk group (Figure 8C), including light yellow module (correlation parameter = 0.68, *p* < 0.001), red module (correlation parameter = 0.5, *p* < 0.001), and brown module (correlation parameter = 0.56, *p* < 0.001, Figures 8D–F). Upregulated DEGs from GSE21653 dataset, genes from three modules, and 86 oncogenes were overlapped and 25 genes were shared in these three groups (Figure 8G). Enrichment analysis showed that cell cycle, progesterone-mediated oocyte maturation, and oocyte meiosis pathways were the top three pathways (Figure 8H). In GO analysis, the top three items enriched in these 25 genes were enzyme binding, ATP binding, and adenyl ribonucleotide binding (Figure 8I). Of the 25 overlapped genes, 9 genes (CDC6, MCM2, MCM4, CDC20, CDK1, BUB1B, PLK1, CCNA2, GINS1) were enriched in cell cycle pathway. Similarly, upregulated DEGs from GSE21653 dataset, genes from three modules, and 51 oncogenes were overlapped and 25 genes were shared in these three groups (Supplementary Figure 6). A total of 13 genes were overlapped and cell cycle was still the main pathway regulated by these 13 genes. Six genes (CDC6 MCM4 CDK1 BUB1B PLK1 CCNA2) were enriched in cell cycle pathway.

## Discussion

Breast cancer poses a great threat to women’s health. Reliable targeted therapy provides prospects for improving the survival of breast cancer. The CRISPR-cas9 screening serves as a cornerstone and is an outstanding way to systematically identify synthetic-lethal genes (Dhanjal et al., 2017). In the current study, we integrated CRISPR-cas9 screening results of breast cancer from DepMap with TCGA dataset, and identified 86 oncogenes that were overexpressed and have a lethal effect on breast cancer. Enrichment analysis revealed that these genes were enriched in cell cycle. Moreover, we established a gene signature screened from 86 genes, and this signature could divide patients into high risk and low risk. Interestingly, cell cycle pathway also ranked the first when we analyzed DEGs between high-risk and low-risk group and genes. Overlap among the 86 oncogenes, DEGs, and WGCNA analysis highlight the cell cycle pathways as well, indicating its importance to breast cancer viability.

In our study, 9 cell cycle related genes mainly involved in G1/S and G2/M phases, and researches on mechanism of these genes have been conducted. MCM2 and MCM4 genes are members of minichromosome maintenance (MCM) protein complex. As a crucial element of the pre-replication complex (pre-RC), they regulate the helicase activity and the formation of the replication forks. Meanwhile, they play an important role in the initiation of DNA replication and unwinding of the DNA strands in the G1 to S transition phase (Forsburg, 2004). High expression of MCM2 was associated with poor survival in breast cancer patients (Samad et al., 2020). In our analysis, we identified that MCM2 and MCM4 are vital to the survival of breast cancer. CDC6 plays an important role in DNA replication and cells could not initiate DNA replication without CDC6 (Borlado and Méndez, 2008). CDC6 helps MCM proteins load onto origins of replication and promotes them to associate with the chromatin. GINS1 also had a vital effect on G1/S transition by tightly interacting with DNA polymerase ε (Pol ε) and participating in DNA replication (Takayama et al., 2003). The rest of the genes mainly had critical function in G2 to M transition. CDK1 is one of the most important proteins that regulate cell cycle progression via associating with cyclin B1 and promote G2 to M transition. High expression of CDK1 was reported in breast cancer compared with normal tissue (Barrett et al., 2002). CDC20 is co-activator of anaphase promoting complex (APC) which is a complex E3 ubiquitin ligase (Foe and Toczyski, 2011). APC^*Cdc*20^ destructs critical cell cycle regulators such as cyclin B, allowing cells to progress from the metaphase to anaphase transition (Kim and Yu, 2011). CDC20 was reported higher expressed in breast cancer and functions as an oncogene, which was also validated in our analysis (Yuan et al., 2006). BUB1B, which blocks the activation of APC^*Cdc*20^, is the central component of the mitotic checkpoint for spindle assembly, and it was proved overexpressed in breast cancer and acts as a oncogene (Koyuncu et al., 2020). PLK1, as a member of polo-like kinase family, is involved in mitotic entry, centrosome maturation, spindle assembly, and cytokinesis process (Golsteyn et al., 1995; García et al., 2020). Results from CRISPR-cas9 screening suggested that knocking out these genes leads to cell death in breast cancer of all subtypes, and inhibitors targeting these genes may be potential therapeutic strategies for breast cancer. Furthermore, upregulation of these genes in breast cancer indicates that they may be good candidates for drug development.

Previous studies constructed a prognostic signature mainly based on the gene expression, while we integrated functional genomic screening with gene expression in the current study. Our gene signature could divide patients into high risk and low risk regardless of the tumor stage. For patients with high risk, appropriate target agent may be prescribed to improve patients’ outcome. The prediction accuracy of our signature was better than classic clinicopathologic parameters such as tumor stage or PAM50 subtype. This signature was the only independent prognostic parameter in multivariate analysis, although subtypes and stage were widely used in clinical practice.

In conclusion, our research systematically studied genes vulnerable to cell viability and cell cycle is a vital pathway to this process. Functional genomic screening was integrated into our gene signature to predict the prognosis of breast cancer, which outperformed classical clinicopathological parameters in prediction accuracy. These cell cycle–related genes may serve as targets for breast cancer therapy.

## Data Availability Statement

The original contributions presented in the study are included in the article/Supplementary Material, further inquiries can be directed to the corresponding author/s.

## Author Contributions

XS participated in all experimental work and drafted the manuscript. XC and KS designed the article. ZW collected the data. All authors contributed to the article and approved the submitted version.

## Conflict of Interest

The authors declare that the research was conducted in the absence of any commercial or financial relationships that could be construed as a potential conflict of interest.

## Footnotes

1 https://depmap.org/portal/download/

2 https://portal.gdc.cancer.gov/

3 http://www.ncbi.nlm.nih.gov/geo/

4 http://string-db.org

## Supplementary Material

Supplementary Figure 1 Mutation rate of 86 genes in TCGA dataset.

Supplementary Figure 2 Kaplan–Meier plot of discovery and validation cohort based on gene signature using cut-off from ROC curve. **(A)** Kaplan–Meier plot of the internal train cohort. **(B)** Kaplan–Meier plot of the internal validation cohort. **(C)** Kaplan–Meier plot of the entire cohort. **(D)** Kaplan–Meier plot of the external validation cohort.

Supplementary Figure 3 Risk plot of the discovery and validation cohort. **(A)** Risk score distribution of patients in the prognostic model in the discovery cohort. **(B)** Relationship between the survival time and risk score rank in the discovery cohort. **(C)** Risk score distribution of patients in the prognostic model in the validation dataset. **(D)** Relationship between the survival time and risk score rank in the validation cohort.

Supplementary Figure 4 Kaplan–Meier plot of risk score in different subtypes. Kaplan–Meier plot of risk score in luminal **(A)** and HER2 **(B)** subtypes in GSE20685 dataset.

Supplementary Figure 5 Kaplan–Meier plot of risk score in different chemotherapy groups. Kaplan–Meier plot of risk score in patients with chemotherapy **(A)** and without chemotherapy **(B)** in GSE20685 dataset.

Supplementary Figure 6 Overlapped genes between three groups. **(A)** Venn diagram of overlapped genes among 51oncogenes, DEGs between high-risk and low-risk patients, and WGCNA modules. **(B)** GO analysis of overlapped genes. **(C)** KEGG analysis of overlapped genes.
