# Supplementary material for: CRISPR-cas9 Screening Identified Lethal Genes Enriched in Cell Cycle Pathway and of Prognosis Significance in Breast Cancer
Source: Front Cell Dev Biol. 2021 Mar 19;9:646774. doi: 10.3389/fcell.2021.646774 (PMC8017240; doi:10.3389/fcell.2021.646774)
Supplement: Supplementary file 1 [file Table_1.DOCX]

| Gene | Coefficient | Gene | Coefficient |
| --- | --- | --- | --- |
| RFC2 | -0.0265293 | NVL | -0.0886184 |
| POLD1 | -0.1598209 | CCNA2 | -0.0512089 |
| MCM6 | 0.46606808 | GINS4 | 0.12279586 |
| ORC1 | -0.1994464 | BUB1B | 0.23230468 |
| TPX2 | 0.05121207 | MIS18A | 0.00434448 |
| PDRG1 | -0.2006849 | DONSON | 0.07936956 |
| ORC6 | 0.11953819 | CHAF1B | -0.0895334 |
| CDC45 | -0.1974628 | TONSL | -0.1993658 |
| CDC6 | 0.03486058 | RACGAP1 | 0.00819347 |
| CDC7 | -0.2349132 | SPC24 | -0.0984569 |
| POLE2 | -0.065393 | POLR3K | -0.0488865 |
| MCM4 | 0.30500568 | CCT3 | -0.0759556 |
| PSMD3 | 0.03573427 | RAD21 | 0.01162862 |
| TIMELESS | 0.14168106 | CENPN | 0.40640201 |
| ECT2 | 0.14890833 | PLK1 | -0.0307446 |
| HSPE1 | 0.00556417 | DTYMK | -0.153131 |
| ATP6V0B | 0.19889644 | FOXD4 | -0.2833653 |
| CENPK | -0.012449 | CDK1 | 0.08712765 |
| SNRPB | -0.1685049 | RRM2 | -0.1330277 |
| ROMO1 | -0.022528 | MTBP | -0.1975694 |
| GINS2 | -0.2077733 | ZNHIT2 | 0.27857906 |
| KIF11 | -0.1604855 | RUVBL1 | 0.01000425 |
| ANAPC11 | 0.26866739 | EXOSC4 | 0.22149092 |
| PFDN2 | -0.0854278 | TRAIP | 0.44723547 |
| DTL | -0.0223717 | PRC1 | 0.00125915 |
| PSMB3 | 0.04256533 |  |  |

Supplementary Table 1. The coefficient of gene signature.
