# Supplementary figures and images for: CRISPR-cas9 Screening Identified Lethal Genes Enriched in Cell Cycle Pathway and of Prognosis Significance in Breast Cancer
Source: Front Cell Dev Biol. 2021 Mar 19;9:646774. doi: 10.3389/fcell.2021.646774 (PMC8017240; doi:10.3389/fcell.2021.646774)

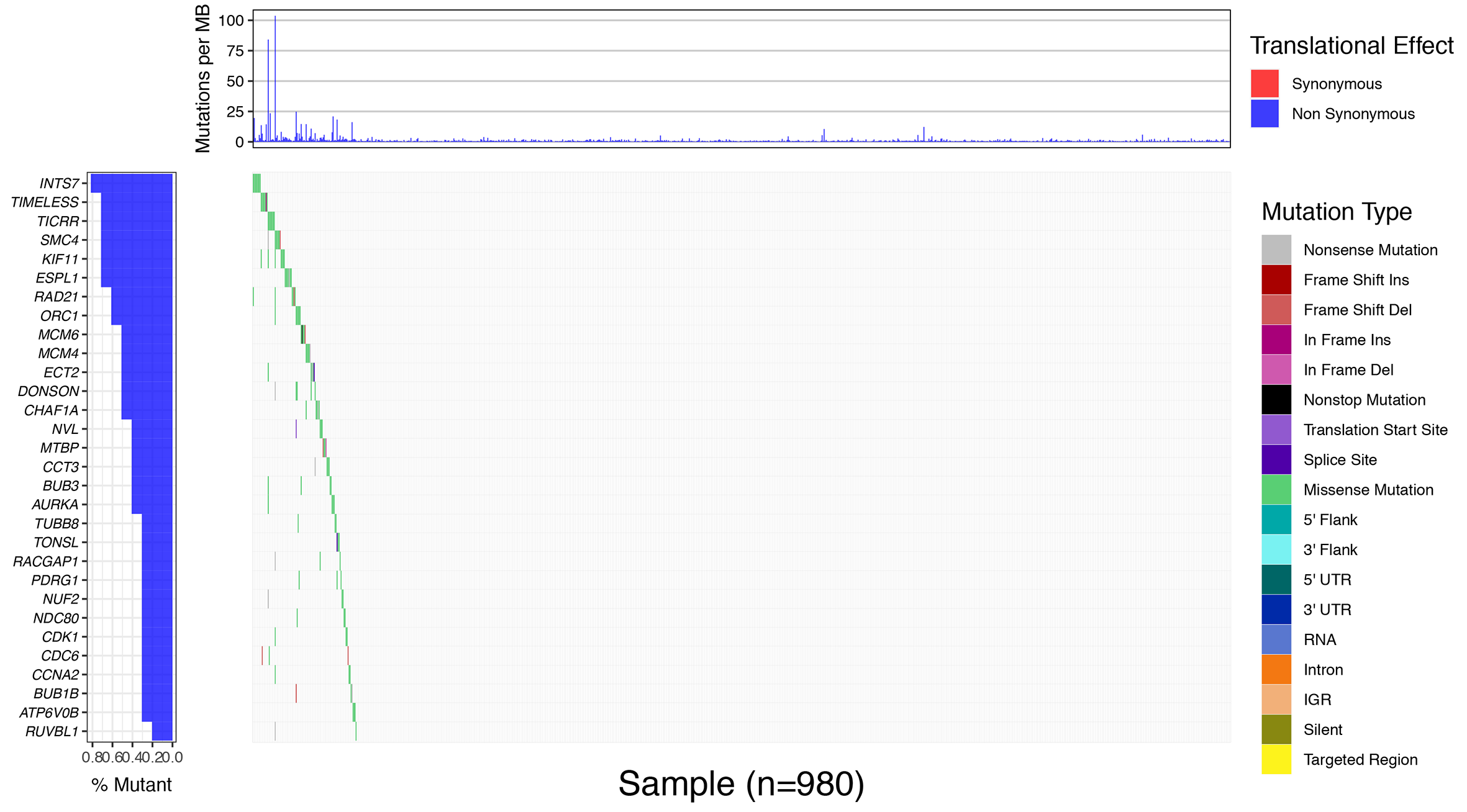

Supplement: Supplementary Figure 1 — Mutation rate of 86 genes in TCGA dataset. [file Image_1.TIF]

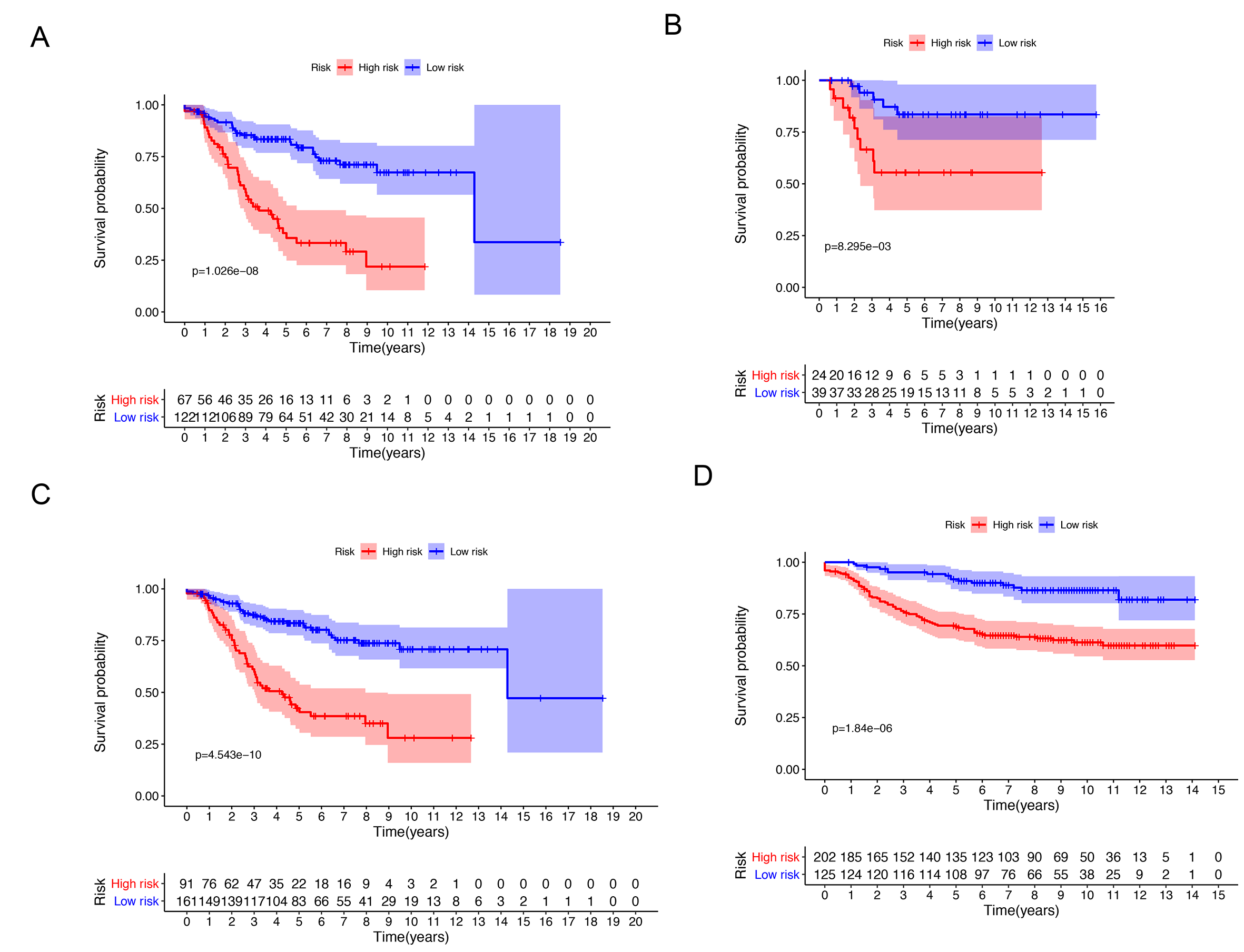

Supplement: Supplementary Figure 2 — Kaplan–Meier plot of discovery and validation cohort based on gene signature using cut-off from ROC curve. (A) Kaplan–Meier plot of the internal train cohort. (B) Kaplan–Meier plot of the internal validation cohort. (C) Kaplan–Meier plot of the entire cohort. (D) Kaplan–Meier plot of the external validation cohort. [file Image_2.TIF]

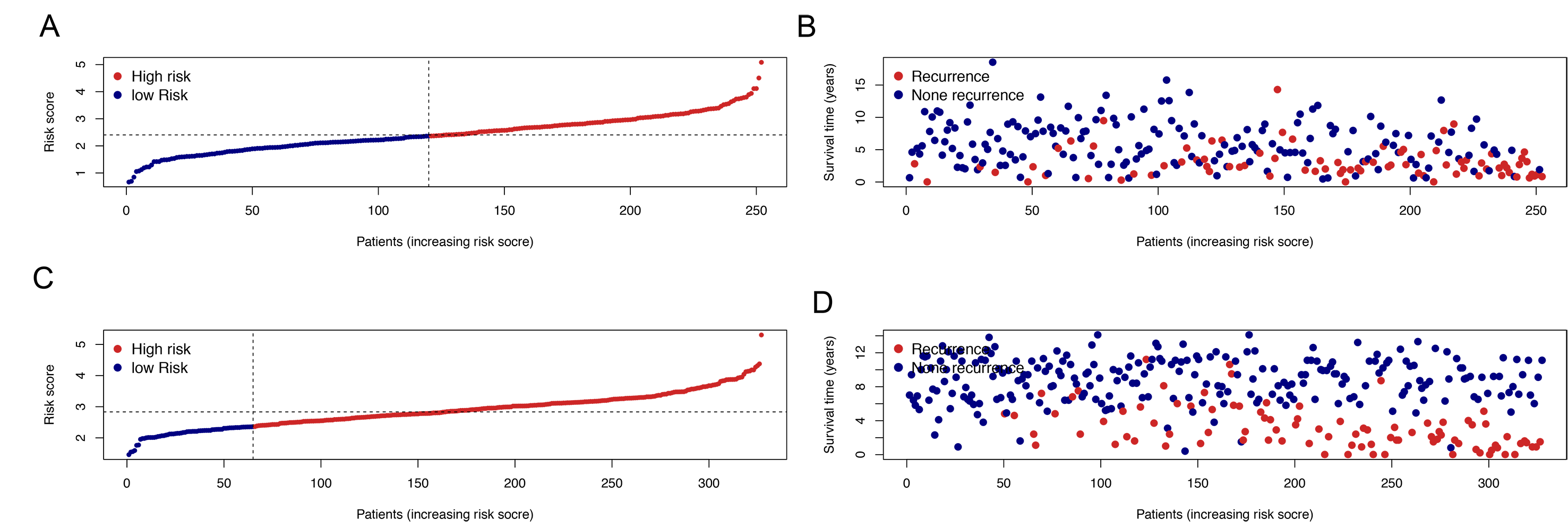

Supplement: Supplementary Figure 3 — Risk plot of the discovery and validation cohort. (A) Risk score distribution of patients in the prognostic model in the discovery cohort. (B) Relationship between the survival time and risk score rank in the discovery cohort. (C) Risk score distribution of patients in the prognostic model in the validation dataset. (D) Relationship between the survival time and risk score rank in the validation cohort. [file Image_3.TIF]

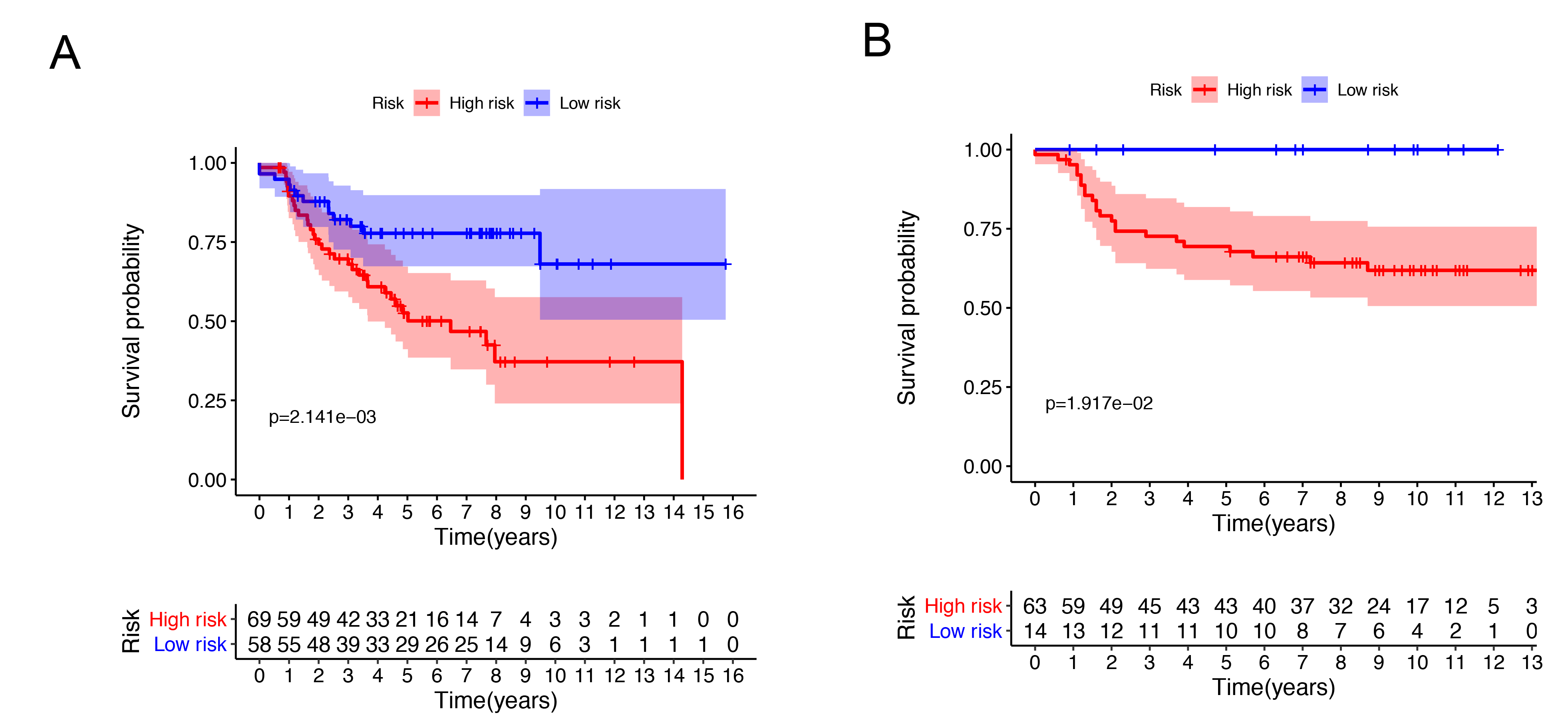

Supplement: Supplementary Figure 4 — Kaplan–Meier plot of risk score in different subtypes. Kaplan–Meier plot of risk score in luminal (A) and HER2 (B) subtypes in GSE20685 dataset. [file Image_4.TIF]

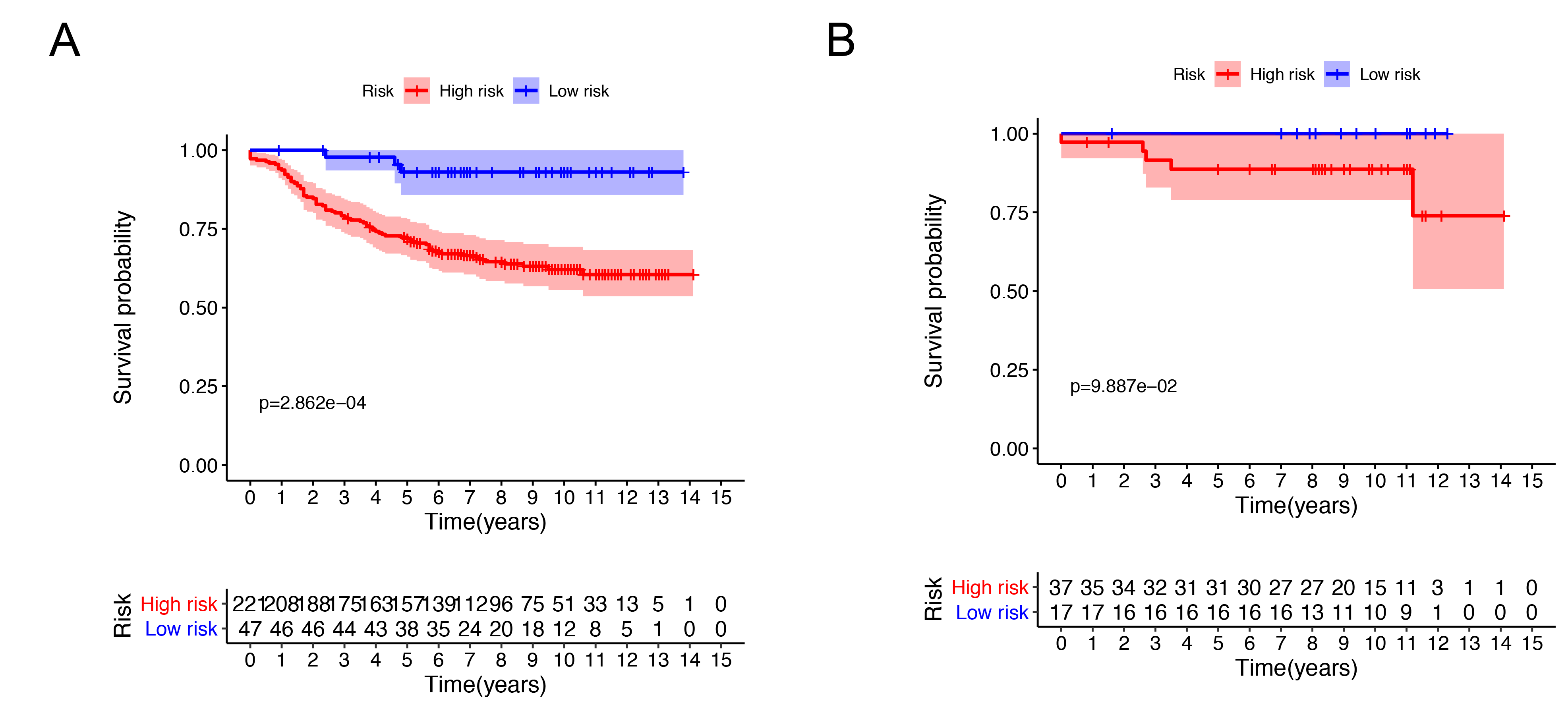

Supplement: Supplementary Figure 5 — Kaplan–Meier plot of risk score in different chemotherapy groups. Kaplan–Meier plot of risk score in patients with chemotherapy (A) and without chemotherapy (B) in GSE20685 dataset. [file Image_5.TIF]

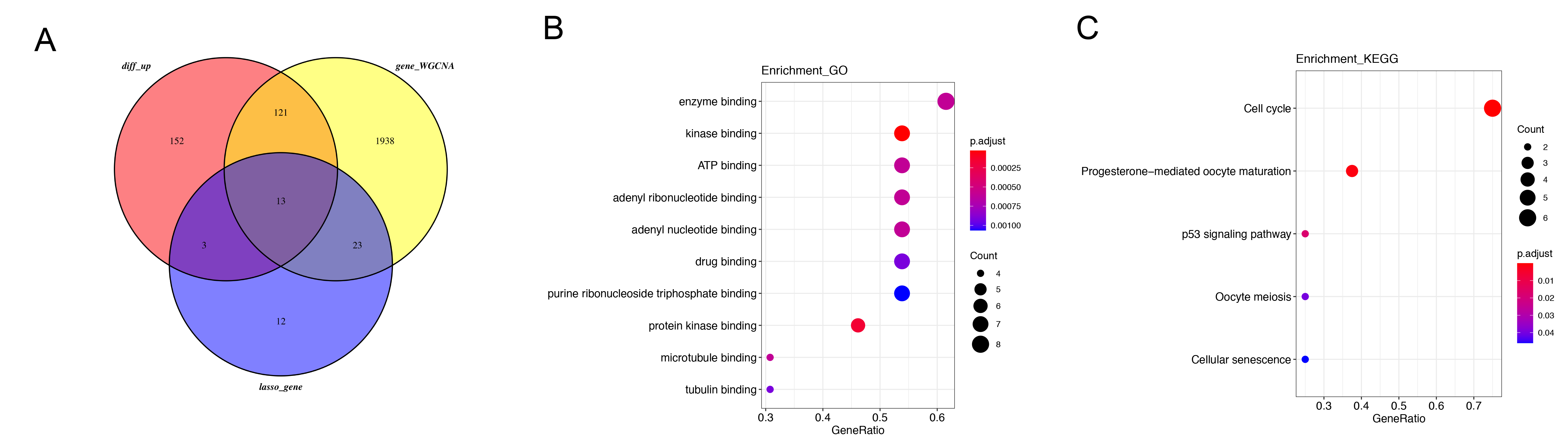

Supplement: Supplementary Figure 6 — Overlapped genes between three groups. (A) Venn diagram of overlapped genes among 51oncogenes, DEGs between high-risk and low-risk patients, and WGCNA modules. (B) GO analysis of overlapped genes. (C) KEGG analysis of overlapped genes. [file Image_6.TIF]
